# Capture efficiency of CONTRAfluran^™^ absorbers for sevoflurane in routine anaesthesia practice: a prospective observational single-center study

**DOI:** 10.1186/s12871-025-03491-3

**Published:** 2025-12-01

**Authors:** Sebastian Gibb, Nadin Möller, Falk von Dincklage, Stephan Knigge

**Affiliations:** Department of Anesthesiology and Intensive Care Medicine, University Medicine Greifswald, Ferdinand-Sauerbruch-Straße, Greifswald, D-17475 Germany

**Keywords:** Balanced anaesthesia, Sevoflurane, Vapour capture technology

## Abstract

**Background:**

Volatile anaesthetics are potent greenhouse gases. Recapturing volatile anaesthetics using active charcoal absorbers on the exhaust port of anaesthesia machines might reduce their negative climate impact. Previous studies investigating the capture efficiency of absorbers have been performed mostly under circumstances that differ from everyday clinical routine. We here investigated the capture efficiency of the CONTRAfluran^™^ absorbers for sevoflurane in routine anaesthesia practice.

**Methods:**

In this prospective single-centre study, we collected 20 consecutive CONTRAfluran^™^ absorbers from two different operating rooms (ORs) after they were maximally saturated. After storage and transport, the sevoflurane was recaptured from the absorbers by the absorber manufacturer. To calculate the capture efficiency, we divided the amount of recaptured sevoflurane by the total sevoflurane consumption during the use of the absorbers, which was obtained by comparing the weight of the sevoflurane bottles before and after refilling the vaporizers.

**Results:**

We monitored 738 balanced anaesthesia cases. A total of 14.2 kg sevoflurane was used and 4.9 kg could be recovered, yielding a capture efficiency of 34%. The capture efficiencies differed significantly between the ORs, with 47.8% in the OR with shorter anaesthesia durations and frequently inhaled inductions versus 21.4% in the OR with longer anaesthesia durations. We saw a 3% weight loss over time during storage.

**Conclusions:**

If CONTRAfluran^™^ absorbers are used until they are fully saturated, almost one-third of the sevoflurane can be recaptured. The capture efficiency may be affected by various patient- and procedure-related factors, including the fresh gas flow used and the duration of anaesthesia.

**Supplementary Information:**

The online version contains supplementary material available at 10.1186/s12871-025-03491-3.

## Background

The anthropogenic climate crisis is a significant threat to global health [1]. The health-care sector is responsible for 5–10% of greenhouse gas emissions [2]. A relevant source of these emissions are volatile anaesthetics (VAs) [2, 3].

Active charcoal absorbers can be attached to the exhaust port of anaesthesia machines to capture VAs and might decrease their emissions. Using absorbers that allow the recapture of the VAs has the additional potential benefit of being able to reuse the captured VAs.

Hinterberg et al. [4] investigated the efficiency of the active charcoal absorbers, CONTRAfluran^™^ anaesthetic gas canisters (ZeoSys medical, Luckenwalde, Germany), for VAs in the operating room (OR). They found that only 25% of the vapourised desflurane could be recaptured. However, as Kalmar et al. pointed out [5], the desorption process to recapture the substances from the canister is highly dependent on the saturation level, with a higher capture efficiency for full, i.e. saturated canisters. Therefore, it expected that a higher efficiency could be obtained, when canisters are used to a higher saturation level than the 15% in the study by Hinterberg et al. [4, 5].

We conducted this exploratory study to investigate the efficiency of passive canisters for sevoflurane and the entire recapture process when canisters for sevoflurane are used to higher saturation levels in a routine anaesthesia practice.

## Methods

### Ethics approval

The study was approved by the ethics committee of the University Medicine Greifswald (Chairperson: Prof. Dr. M. Dörr; reference number: BB 102/23; approval date: 8 August 2023).

### Definitions

We apply the taxonomy recently introduced by Gandhi et al. [6]. Briefly, the capture efficiency refers to the entire recapture process and includes the mass of VA that was used in comparison to the mass that was eventually recovered from the canister and could be reused. Proportional capture efficiency is the calculated efficiency for each individual canister. The in vivo mass transfer describes the increase in mass of the canister in proportion to the mass of the VA used in a clinical setting.

### Setting

We collected a total of 20 consecutive CONTRAfluran™ canisters in two different ORs in this prospective single-centre exploratory observational study.

The first OR (referred to as OR1), was dedicated to ear, nose and throat surgery. Here, a median of five cases per day were anaesthetized and then generally managed with a supraglottic airway. All cases were induced and emerged from anaesthesia in the OR. The first and second case each day were generally children, where we did inhaled inductions with a fresh gas flow in the range of the minute ventilation of the respective child. The following cases in that OR were mostly adults.

The second OR (referred to as OR2) was dedicated to spine surgery. Here, the median number of cases was two per day and the patients were generally adults who were intubated. The first case was always induced in the OR, the following cases rarely in a special induction room where no canister was installed. All cases emerged from anaesthesia in the OR.

All anaesthesia cases were managed at the discretion of the anaesthesiologists. Following induction, a minimal or metabolic fresh gas flow (< 0.5 l·min^−1^, mostly 0.3 l·min^−1^) was used during the steady state phase in all cases. We did not record patient data, type of anaesthesia, induction strategy or airway used due to the exploratory, pragmatic and anonymous nature of the study.

We used the Draeger connect software (Draeger Medical Deutschland GmbH, Luebeck, Germany) to determine the number, duration and type of anaesthesia cases.

### Consumption and weight measurement

Balanced anaesthesia with VAs was given using Draeger Perseus A500 anaesthesia machines equipped with bypass vaporizers of the type Draeger D-Vapor 3000 (Draeger Medicine Deutschland GmbH, Luebeck, Germany), and a calcium and sodium hydroxide-based carbon dioxide absorber of the type Draegersorb CLIC 800+ (Draeger Medicine Deutschland GmbH, Luebeck, Germany). The canisters were permanently connected to the exhaust port of the anaesthesia machines, which work in a passive mode, without an AGSS connected. We weighed the sevoflurane (SEVOrane, AbbVie Deutschland GmbH & Co. KG, Mainz, Germany) bottles before and after maximum filling of the vaporizer and used the difference in weight to determine the sevoflurane consumption. The vaporizers were filled to the maximum before we attached the first CONTRAfluran™ canister (ZeoSys medical, Luckenwalde, Germany). During the study period, almost every day the two vaporizers were filled completely and the sevoflurane bottle weight difference measured by the study team (NM and SK), which were not involved in providing the anaesthesia.

In the case where a new bottle of sevoflurane was opened, it was initially attached to a non-study vaporizer to relieve any excess pressure, and was weighed before the study vaporizer was filled.

Each canister was only changed after it had been exhausted as identified by a visible red LED and an acoustic warning signal from the SENSOfluran^™^ sensor unit (ZeoSys medical, Luckenwalde, Germany). Subsequently, we refilled the vaporizer to its maximum and measured the weight difference of the sevoflurane bottle, again. The weight difference was counted as consumption for the just exhausted canister.

The weight gain of a canister was determined by weighing the canister before usage and after it had been exhausted to determine the in vivo mass transfer (ratio of absorber weight gain to consumed weight of sevoflurane). Each exhausted canister was recapped and stored in a closed plastic zip-lock bag.

ZeoSys needs at least 20 canisters for a single desorption process. Before returning the canisters to ZeoSys, we weighed all canisters again to determine any potential weight loss.

All weight measurements were done with a precision scale (Kern PCB 2500-2; Kern&Sohn GmbH, Balingen-Frommern; Germany; maximum weight = 2500 g; d = 0.01 g).

ZeoSys stored all canisters for at least four weeks, due to regulatory reasons. Before starting the desorption and recovery process they weighed all canisters (Sartorius Cubis II MCA5201S-2S00-0; Sartorius AG, Göttingen; Germany; maximum weight = 5200 g; d = 0.1 g). Afterwards, they sent us the information about the total amount of sevoflurane recovered from all 20 canisters.

The capture efficiency was then determined by calculating the total amount of sevoflurane recovered relative to the amount used [6]. To obtain the proportional capture efficiency, we multiplied the total amount of sevoflurane recovered by the ratio of the individual weight gain of each canister to the total weight gain of all canisters.

### Outcome

The primary outcome was the amount of recovered sevoflurane reported by ZeoSys. The secondary outcomes were the difference between the in vivo mass transfer, the recapture rate reported by ZeoSys, the difference in the sevoflurane recovery between both ORs and the weight loss of the canister during storage.

### Data processing and statistical analysis

All data processing and statistical analyses were performed using R version 4.4.0 [7]. The two-sided Wilcoxon rank-sum test was used for statistical comparison. A *p*-value less than 0.05 was considered to be statistically significant. Summary tables were generated using the *gtsummary* package [8]. All data and analysis can be found in the zenodo repository, 10.5281/zenodo.10442174 [9].

## Results

### Sevoflurane consumption and capture efficiency

We monitored a total of 968 anaesthesia cases during the 26 week study period, of which 76% received VAs for a total anaesthesia duration of 1348 h.

A total of 14.2 kg sevoflurane was used and 4.9 kg could be recaptured (capture efficiency 34%). Sevoflurane attributed 63.4% of the canisters’ weight gain. The remaining 36.6% were water (and only traces of metabolic products like acetone, Compound A-E, etc.).

Comparing the two ORs, we found a large and significant difference in the total sevoflurane used, in the ratio of patients receiving VAs and the durations of anaesthesia (Table 1A and B).

While the median weight gain of the canisters did not differ between the two ORs, we observed a large and significant difference in the in vivo mass transfer, which was at 78.1% for OR1 and 34.9% for OR2 due to the significantly different sevoflurane consumption in the two ORs (Table 1B and C). Thus, assuming the same proportion of sevoflurane could be accounted for the relative weight gain of each canister, the proportional capture efficiency differed significantly between the two ORs, with 47.8% for OR1 and 21.4% for OR2 (Table 1C).

**Table 1:** Characteristics of all 20 anaesthetic gas canisters. cases, durations, sevoflurane consumption, etc. Are given as per canister. Values Are median (lower and upper quartile); p-values Are calculated using the Wilcoxon rank sum (exact) test. ENT: ear, nose and throat surgery

Group	Characteristics per canister	OR1 (ENT), *N* = 15 canisters	OR2 (Neurosurgery), *N* = 5 canisters	*p*-value
(A) Case summary	Total number of cases	43.5 (37.0, 50.3)	70.2 (59.6, 75.5)	0.011
	Number of inhaled anaesthesia cases	28.0 (26.3, 34.0)	64.2 (55.6, 68.5)	0.002
	Total duration of inhaled anaesthesia [h]	32.1 (30.4, 36.3)	168.0 (141.6, 191.0)	< 0.001
	Average duration of inhaled anaesthesia [min]	69.3 (62.2, 74.5)	181.2 (178.6, 181.2)	< 0.001
(B) Sevoflurane consumption	Total sevoflurane weight used [g]	524.9 (483.4, 559.2)	1,261.4 (1,070.0, 1,271.3)	< 0.001
	Average sevoflurane weight used per hour inhaled anaesthesia [g·h^− 1^]	15.8 (14.3, 17.5)	7.5 (6.8, 7.8)	< 0.001
(C) Canister characteristics	Weight gain [g]	399.0 (385.8, 415.7)	381.0 (373.0, 384.5)	0.13
	In vivo mass transfer [%]	78.1 (72.0, 81.1)	34.9 (27.5, 35.6)	< 0.001
	Proportional sevoflurane captured [g]	244.6 (236.5, 254.8)	233.6 (228.6, 235.7)	0.13
	Proportional capture efficiency [%]	47.8 (44.1, 49.7)	21.4 (16.9, 21.8)	< 0.001

The details of each canister can be found in Supplemental Table S1.

### Weight loss

**Fig. 1:**
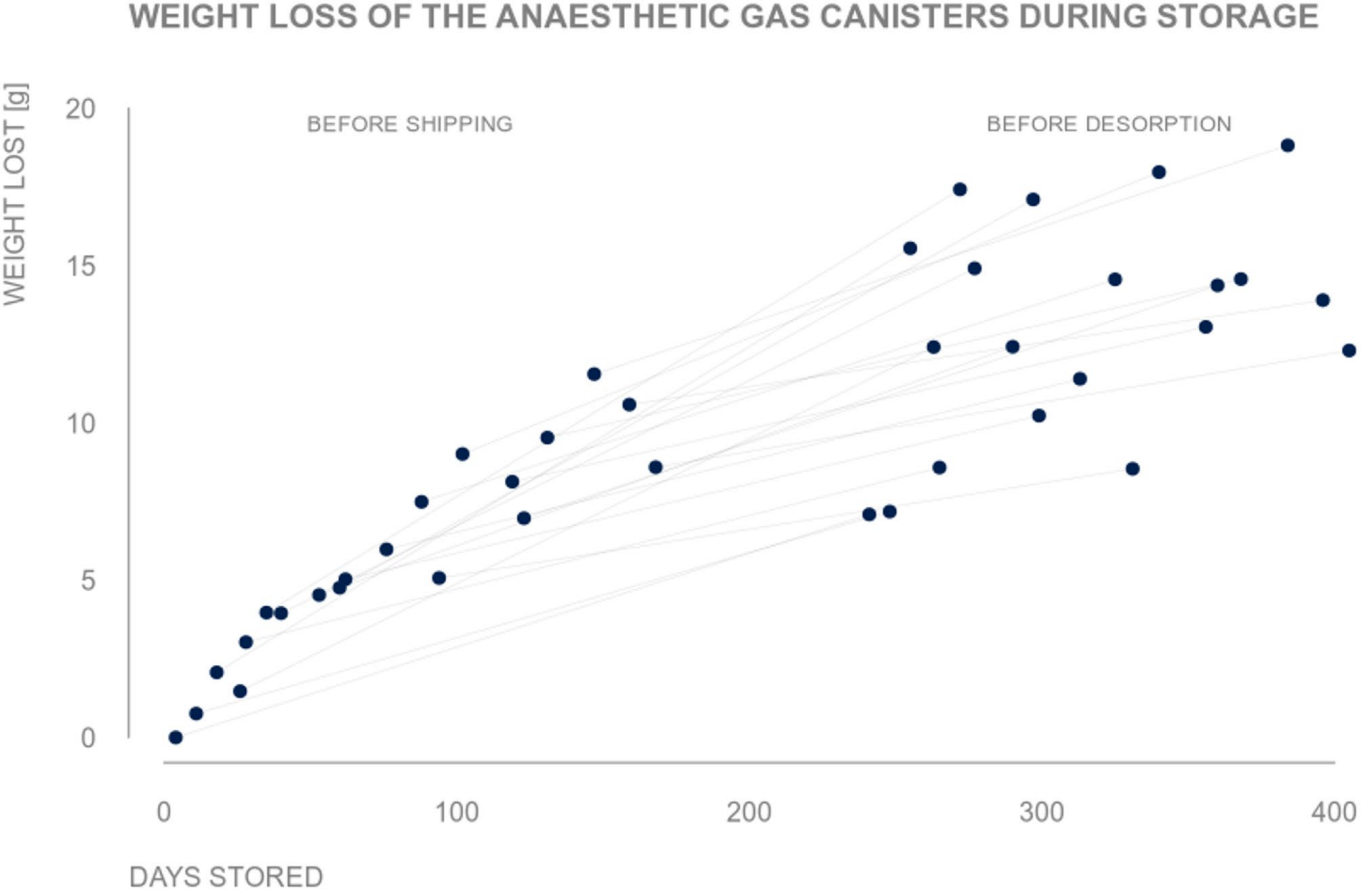
Weight loss of the anaesthetic gas canisters during storage. The dots represent the weight loss of each individual anaesthetic gas canister for the days after exhaustion before shipping and desorption. The gray lines connect the same anaesthetic gas canisters measured at the two different time points

The 20 saturated canisters were stored for 69.0 (33.2, 120.0) days in median (lower and upper quartile) in our hospital and additional 237 days at ZeoSys before the desorption process. Over the entire storage period we observed a loss of 13.5 (11.1, 15.1) gram in median (lower and upper quartile) for each canister and of 262.37 gram in total. This corresponds to 3.3% of the canisters’ weight gain (Fig. 1).

## Discussion

We demonstrated in this prospective single-center observational study that using active charcoal-based canisters for sevoflurane until they reached their maximum saturation levels can yield a total capture efficiency of 34%. This represents 4.9 kg of sevoflurane that can be recovered from a total of 14.2 kg sevoflurane used in our study cohort of 738 balanced anaesthesia cases.

Interestingly, we saw a large and significant difference in the proportional capture efficiencies in the two ORs, with 47.8% for OR1 and 21.4% for OR2. As OR1 had a higher total number of inductions and far more inhaled inductions, a higher wastage of sevoflurane during the induction phase could be a major cause for this difference. During an inhaled induction even if done with a fresh gas flow in the range of the minute ventilation of the respective child, a large amount of sevoflurane passes the patients without uptake, thus increasing the relative amount that can be captured by the canister and yielding higher capture efficiency in OR1.

Another determinant for the lower capture efficiency in OR2 may be the longer duration of anaesthesia compared to OR1. Hinterberg et al. had also recognised a much lower capture efficiency in longer rather than shorter anaesthesia [4]. In shorter anaesthesia cases, the typically higher and therefore more wasteful fresh gas flow during induction contribute to the higher capture efficiency, which is reflected in the higher average VA consumption per hour in OR1 (Table 1B). During maintenance the vented sevoflurane does not vary substantially, yielding a nearly constant mass transfer (if the fresh gas flow and the vaporizer setting is lowered accordingly) [10, 11].

Our overall capture efficiency of 34% is similar to the 25% for desflurane [4] and the 43–51% previously reported for the AGSS-dependent SageTech Medical’s Volatile Capture Device (SageTech Medical, Paignton, UK; [12]) or the 45% in vivo mass transfer for CONTRAfluran^™^ during laparoscopic surgery [13]. Hinterberg et al. used a new canister for every case, which produced underfilled canisters with less than 15% of their capacity, possibly yielding a relative higher contribution of water to the weight gain and insufficiencies in the desorption and recapture process [4, 5]. By contrast, our study represents a routine anaesthesia practice usage of the canisters until they were saturated and also incorporates the loss during storage.

Despite the difference in the overall capture efficiency, our results in the different ORs confirm that a longer duration of minimal-flow anaesthesia reduces the capture efficiency, as described and simulated previously [4, 10, 11]. However, the focus on the capture efficiency as a target is misleading as a higher fresh gas flow yields a higher capture efficiency due to an increased proportion of anaesthetic wasted and entering the canister [6, 11, 14].

The absorption of the VA in the canister is based on strong non-covalent interactions displacing water [11]. In our study, 63.4% was attributable to sevoflurane. This is in line with the 70% reported previously for CONTRAfluran^™^ [4]. Perhaps a connected AGSS caused a dryer canister, which could be a reason for the very high desorption efficiency of 95.5% sevoflurane for SageTech’s AGSS-dependent volatile capture device [12].

Although we stored all canisters in a zip lock bag, we found a decrease in weight of around 3% during storage (Fig. 1). In contrast a previous study reported a constant weight over time [4]. We could not ascertain whether this was due to water evaporation or spontaneous VA desorption.

The weight loss, the unknown contribution of water and metabolic products, and the difference in desorption efficiency illustrate that it is not possible to draw any conclusions about the overall capture efficiency of canisters from the (in vivo) mass transfer alone.

### Limitations

The primary limitation of our study is that we included only two ORs with a different number of patients and a variety of surgical and anaesthesia procedures. Due to the large number of patients per canister, the long observation period and the pragmatic design, we were not able to analyse patient- or procedure-related factors like fresh gas flow, minute ventilation, MAC value, airway devices. Therefore, our findings in these two ORs cannot be generalised and should be regarded as exploratory, indicating a further need for research.

The canisters were permanently connected to the exhaust port of the anaesthesia machines, as it is the common practice in most ORs. This may have lowered our capture efficiency due to spontaneous desorption from the canisters and due to periods with higher fresh gas flows, such as induction, emergence, or total intravenous anaesthesia where flow-dependent desorption may occur [15, 16].

We calculated the proportional capture efficiency based on the simplified assumption that the sevoflurane-to-water ratio is the same in all canisters. However, the sevoflurane binding capacity of activated carbon can be significantly reduced by water [17]. Thus, a higher humidity caused by the lower fresh gas flows in OR2 may also have contributed to the lower capture efficiency in this OR.

Seven of 328 cases of OR2 were induced in a separate induction room. This may slightly under- or overestimate the proportional capture efficiency of the canisters in this OR because the wasteful induction was not captured and/or an external, unmeasured amount of sevoflurane may be introduced. The intraoperative change of the carbon dioxide absorber was infrequent and should be negligible.

We decided to weigh the sevoflurane bottles instead of the vaporizer to not interrupt the anaesthesia process by removing/installing the vaporizer during our measurements and to avoid transporting the vaporizers or the scale into different ORs. We validated this method and found a high agreement with a mean difference of −0.07 g (Supplemental Section S2 and Fig. S1). By weighing the sevoflurane bottles, we measure all, possibly hidden, losses of VAs (e.g. spilled sevoflurane, losses of the vaporizer) and the accuracy is enhanced by using a smaller more precise scale.

After the removal of the last exhausted canister, CH0100012088, in OR2 the vaporizer was not refilled and the weight difference of the sevoflurane bottle not recorded. Instead we calculated the sevoflurane consumption data for the four last anaesthesia cases as described by Biro et al. (Supplemental Section S3) [18]. This may slightly underestimate the in vivo mass transfer and proportional capture efficiency.

ZeoSys needs at least 20 canisters for a single desorption process. Therefore, we chose to collect the minimum number of 20 consecutive canisters. A larger number of canisters would have meant significantly more effort (collecting 20 canisters in two ORs already took over 6 months and almost 1000 patients) without a relevant higher information value. However, a regular desorption process requires 80 canisters. During the desorption process, the various components of the captured gas are separated according to their boiling points and densities. After cooling, the compounds form liquid layers, with sevoflurane at the bottom due to its high density. To remove by-products such as acetone and compounds A–E, a specific amount of the supernatant is removed. Therefore, our desorption efficiency and, thus, our capture efficiency is slightly lower due to a slightly less efficient process compared to a regular 80 canisters-desorption process.

## Conclusion

The capture efficiency of active charcoal-based canisters (CONTRAfluran™) might depend on various patient- and procedure-related factors, including the fresh gas flow used and the duration of anaesthesia. Using metabolic fresh gas flow, the proportional capture efficiencies were 47.8% and 21.4%, respectively, in the two ORs studied. However, focusing on capture efficiency is misleading. Further research and independent life cycle analyses are needed to understand the factors of different capture efficiencies and determine the carbon footprint of balanced anaesthesia with vapour capture technology.

## Supplementary Information

Supplementary Material 1.

## Data Availability

The datasets generated and/or analysed during the current study are available in the zenodo repository, 10.5281/zenodo.10442174 [9].
