# Supplementary material for: Capture efficiency of CONTRAfluran™ absorbers for sevoflurane in routine anaesthesia practice: a prospective observational single-center study
Source: BMC Anesthesiol. 2025 Dec 1;25:600. doi: 10.1186/s12871-025-03491-3 (PMC12670840; doi:10.1186/s12871-025-03491-3)
Supplement: Supplementary file 1 — Supplementary Material 1. [file 12871_2025_3491_MOESM1_ESM.docx]

Supplement - Capture efficiency of CONTRAfluran^TM^ absorbers for sevoflurane in routine anaesthesia practice: a prospective observational single-center study

# S1 Table of anaesthetic gas canisters

Table S1: Details of anaesthetic gas canisters. C_sev_: total sevoflurane used [g]; C_avg_: average sevoflurane used [g·h^-1^]; D: total duration [h]; D_sev_: total duration of sevoflurane cases [h]; D_avg_: average duration of sevoflurane cases [h]; Id: lot number; MT: in vivo mass transfer [%]; N: total number of anaesthesia cases; N_sev_: total number of sevoflurane cases; OR: operation room; S: days stored after exhausted before shipping; Z: days stored before desorption; W_d_: weight gain; W_f_: final weight when exhausted, directly after disconnection; W_i_: initial weight; W_l_: weight lost after exhausted and before shipping; W_z_: weight lost just before desorption; ^*^: the sevoflurane consumption of 54.10 g for the last four cases was estimated (7.3).

| **Id** | **OR** | **W_i_** | **W_f_** | **W_d_** | **W_l_** | **W_z_** | **S** | **Z** | **N** | **N_sev_** | **D** | **D_sev_** | **D_avg_** | **C_sev_** | **C_avg_** | **MT** |
| --- | --- | --- | --- | --- | --- | --- | --- | --- | --- | --- | --- | --- | --- | --- | --- | --- |
| CH0100009185 | OR1 | 1031.0 | 1456.8 | 425.8 | 9.0 | 18.0 | 102 | 340 | 52.0 | 41.0 | 3702 | 2842 | 69.3 | 610.7 | 12.9 | 69.7 |
| CH0100009250 | OR1 | 1065.5 | 1488.8 | 423.3 | 4.0 | 14.9 | 40 | 277 | 46.1 | 29.8 | 3802 | 1870 | 62.8 | 524.9 | 16.8 | 80.7 |
| CH0100009255 | OR2 | 1048.6 | 1433.2 | 384.5 | 2.1 | 15.6 | 18 | 255 | 81.9 | 76.9 | 14824 | 13932 | 181.2 | 1807.2 | 7.8 | 21.3 |
| CH0100009267 | OR1 | 1047.4 | 1472.9 | 425.6 | 4.5 | 12.4 | 53 | 290 | 39.1 | 27.0 | 2179 | 1485 | 55.0 | 543.4 | 22.0 | 78.3 |
| CH0100009273 | OR1 | 1047.6 | 1438.4 | 390.8 | 0.8 | 7.2 | 11 | 248 | 52.0 | 28.0 | 3155 | 1953 | 69.8 | 500.7 | 15.4 | 78.1 |
| CH0100012066 | OR1 | 1028.4 | 1415.2 | 386.8 | 9.5 | 14.6 | 131 | 368 | 49.5 | 34.0 | 2708 | 1928 | 56.7 | 471.1 | 14.7 | 82.1 |
| CH0100012067 | OR1 | 1028.7 | 1425.9 | 397.3 | 1.5 | 12.4 | 26 | 263 | 49.6 | 34.1 | 3207 | 2251 | 66.1 | 555.9 | 14.8 | 71.5 |
| CH0100012074 | OR1 | 1022.8 | 1407.2 | 384.4 | 8.1 | 13.1 | 119 | 356 | 51.0 | 35.0 | 3481 | 2218 | 63.4 | 584.1 | 15.8 | 65.8 |
| CH0100012076 | OR1 | 1029.3 | 1437.2 | 407.9 | 4.0 | 17.4 | 35 | 272 | 27.7 | 21.7 | 2225 | 1876 | 86.6 | 562.5 | 18.0 | 72.5 |
| CH0100012088^*^ | OR2 | 1034.0 | 1383.6 | 349.5 | 0.0 | 7.1 | 4 | 241 | 70.2 | 64.2 | 12592 | 11458 | 178.6 | 1271.3 | 6.7 | 27.5 |
| CH0100012089 | OR1 | 1034.2 | 1415.2 | 381.0 | 10.6 | 13.9 | 159 | 396 | 35.0 | 27.0 | 2395 | 1913 | 70.9 | 514.1 | 16.1 | 74.1 |
| CH0100012091 | OR1 | 1024.6 | 1434.6 | 410.0 | 11.6 | 18.8 | 147 | 384 | 29.9 | 20.9 | 2437 | 1780 | 85.1 | 533.2 | 18.0 | 76.9 |
| CH0100012196 | OR1 | 1020.0 | 1404.9 | 384.9 | 8.6 | 12.3 | 168 | 405 | 31.6 | 19.6 | 2000 | 1194 | 60.8 | 473.3 | 23.8 | 81.3 |
| CH0100012198 | OR1 | 1022.2 | 1401.8 | 379.6 | 3.0 | 8.6 | 28 | 265 | 51.4 | 34.4 | 3637 | 2595 | 75.5 | 577.6 | 13.4 | 65.7 |
| CH0100022360 | OR1 | 1039.1 | 1438.1 | 399.0 | 6.0 | 11.4 | 76 | 313 | 39.0 | 29.0 | 3152 | 2132 | 73.5 | 493.5 | 13.9 | 80.8 |
| CH0100022366 | OR2 | 1028.4 | 1469.2 | 440.8 | 4.8 | 17.1 | 60 | 297 | 59.6 | 55.6 | 10767 | 10079 | 181.2 | 1261.4 | 7.5 | 34.9 |
| CH0100022367 | OR1 | 1040.0 | 1461.5 | 421.4 | 7.5 | 14.6 | 88 | 325 | 42.5 | 26.5 | 3303 | 1635 | 61.6 | 465.0 | 17.1 | 90.6 |
| CH0100022433 | OR2 | 1031.8 | 1404.8 | 373.0 | 7.0 | 14.4 | 123 | 360 | 75.5 | 68.5 | 9529 | 8497 | 124.0 | 957.6 | 6.8 | 38.9 |
| CH0100022435 | OR2 | 1043.8 | 1424.8 | 381.0 | 5.1 | 8.5 | 94 | 331 | 40.9 | 38.9 | 7685 | 7189 | 184.9 | 1070.0 | 8.9 | 35.6 |
| CH0100022442 | OR1 | 1034.9 | 1441.3 | 406.4 | 5.0 | 10.2 | 62 | 299 | 43.5 | 26.0 | 2721 | 2031 | 78.1 | 462.2 | 13.7 | 87.9 |

# S2 Comparing sevoflurane consumption measurements

We weighed the sevoflurane (SEVOrane, AbbVie Deutschland GmbH & Co. KG, Mainz, Germany) bottles before and after filling of the Draeger D-Vapor 3000 (Draeger Medicine Deutschland GmbH, Luebeck, Germany) vaporizer and record the difference. Subsequently we determine the difference using the gold standard - weighing the vaporizer before and after filling. The bottles and the vaporizers were weighed with a Kern PCB 2500-2 scale (Kern&Sohn GmbH, Balingen-Frommern; Germany; maximum weight = 2500 g; d = 0.01 g) and a Kern PNJ 12000-M1 scale (Kern&Sohn GmbH, Balingen-Frommern; Germany; maximum weight = 12000 g; d = 0.1 g), respectively.

We did 100 comparisons (400 weight measurements in total) and found a mean difference of -0.07 g (95 % CI: -0.17, 0.03). The 95 % limits of agreement are -1.08 to 0.94 g.


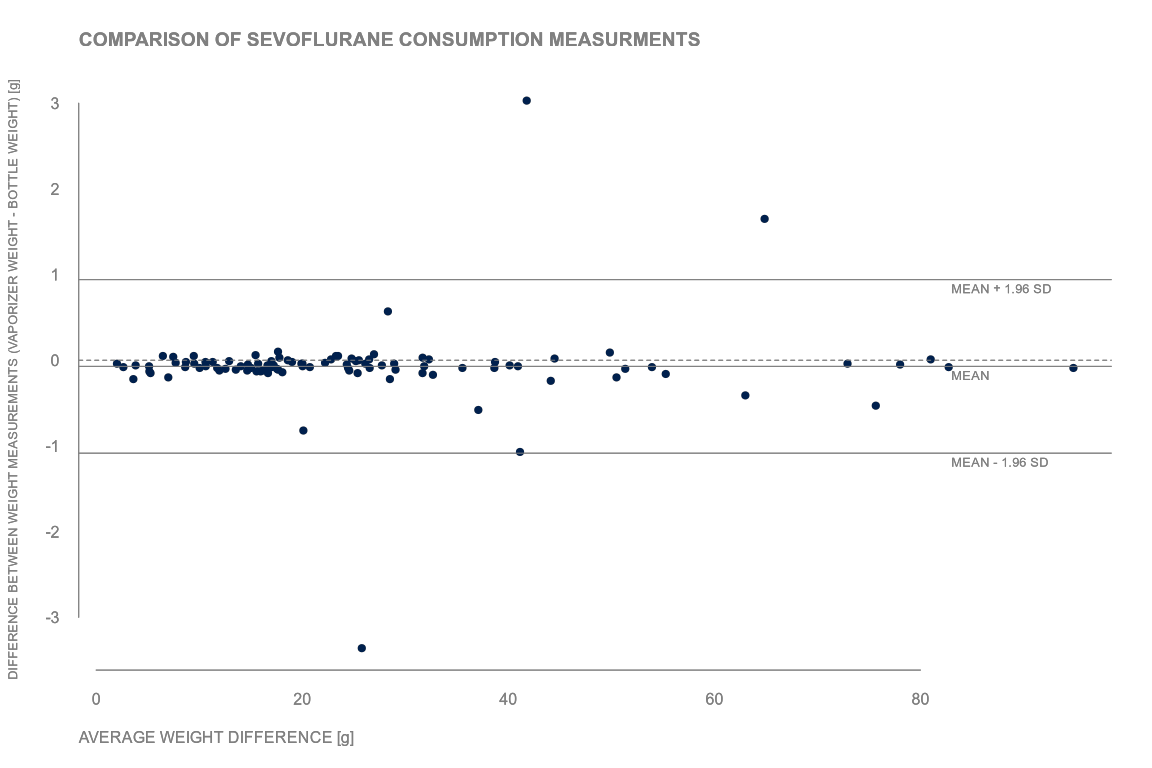


Figure S1: Bland-Altman plot comparing weight methods for determination of sevoflurane consumption.

# S3 Estimation of sevoflurane consumption

After changing the last anaesthetic gas canister *CH0100012088* in OR2, the vaporizer was not refilled. Therefore, we retrospectively calculated the sevoflurane consumption for the last four anaesthesia cases utilising the user log entry provided by the Draeger Perseus anaesthesia machine. The calculation was done as described in Biro et al. with the following formula [[17](#ref-Biro2015)].

$$agentconsumption\left[ g \right)=\frac{freshgasflow\left[ mL\cdot min^{-1} \right)*vaporsetting\left[ Vol\text{\%} \right)*anaesthesiaduration\left[ min \right)}{saturatedgasvolume\left[ mL \right)*100\left[ Vol\text{\%} \right)}*density\left[ g\cdot mL^{-1} \right)$$

We used 184 mL as *saturated gas volume* [[17](#ref-Biro2015)] and a density of 1.52 g·mL^-1^ for sevoflurane [[2](#ref-Hu2021)].

These four cases yield a calculated consumption of 54.10 g sevoflurane. Biro et al. described an overestimation of the sevoflurane consumption by 6.2 %. This would result in an error of 0.26 % for our anaesthetic gas canister CH0100012088 and just slightly underestimate the *in vivo* mass transfer and proportional capture efficiency.

Table S2: Overview of settings and consumption of the last four cases for anaesthetic gas canister *CH0100012088*.

| Case | Time since vaporizer opened [min] | Fresh gas flow [l·min^-1^] | Vaporizer setting [Vol%] | Duration [min] | Sevoflurane consumption [g] |
| --- | --- | --- | --- | --- | --- |
| 1 | 0 | 1.0 | 3.31 | 2 | 0.55 |
| 1 | 2 | 1.0 | 5.09 | 1 | 0.42 |
| 1 | 3 | 0.3 | 8.00 | 5 | 0.99 |
| 1 | 8 | 0.3 | 6.11 | 6 | 0.91 |
| 1 | 14 | 0.3 | 5.14 | 1 | 0.13 |
| 1 | 15 | 0.2 | 5.14 | 2 | 0.17 |
| 1 | 17 | 0.2 | 3.52 | 38 | 2.21 |
| 1 | 55 | 0.2 | 4.48 | 3 | 0.22 |
| 1 | 58 | 0.2 | 5.00 | 2 | 0.17 |
| 1 | 60 | 0.2 | 6.11 | 55 | 5.55 |
| 1 | 115 | 0.2 | 4.57 | 29 | 2.19 |
| 2 | 0 | 0.2 | 8.00 | 13 | 1.72 |
| 2 | 13 | 0.2 | 6.70 | 1 | 0.11 |
| 2 | 14 | 0.2 | 5.04 | 2 | 0.17 |
| 2 | 16 | 0.2 | 3.56 | 6 | 0.35 |
| 2 | 22 | 0.3 | 4.06 | 11 | 1.11 |
| 2 | 33 | 0.3 | 2.57 | 1 | 0.06 |
| 2 | 34 | 0.3 | 1.49 | 1 | 0.04 |
| 2 | 35 | 0.3 | 0.62 | 9 | 0.14 |
| 2 | 44 | 0.3 | 2.50 | 11 | 0.68 |
| 2 | 55 | 0.3 | 3.96 | 8 | 0.79 |
| 2 | 63 | 0.3 | 3.05 | 49 | 3.71 |
| 2 | 112 | 0.3 | 2.93 | 19 | 1.38 |
| 2 | 131 | 0.2 | 2.93 | 5 | 0.24 |
| 2 | 136 | 0.3 | 2.93 | 54 | 3.92 |
| 2 | 190 | 0.3 | 1.53 | 3 | 0.11 |
| 2 | 198 | 0.3 | 1.75 | 4 | 0.17 |
| 2 | 202 | 0.3 | 4.01 | 29 | 2.88 |
| 3 | 0 | 15.0 | 3.56 | 1 | 4.41 |
| 3 | 1 | 0.2 | 3.56 | 9 | 0.53 |
| 3 | 10 | 0.2 | 8.00 | 14 | 1.85 |
| 3 | 24 | 0.2 | 5.04 | 1 | 0.08 |
| 3 | 25 | 0.3 | 5.04 | 8 | 1.00 |
| 3 | 33 | 0.3 | 3.65 | 3 | 0.27 |
| 3 | 36 | 0.3 | 2.54 | 12 | 0.76 |
| 3 | 48 | 0.3 | 3.56 | 7 | 0.62 |
| 3 | 55 | 0.3 | 4.53 | 4 | 0.45 |
| 3 | 59 | 0.3 | 5.58 | 17 | 2.35 |
| 3 | 76 | 0.3 | 3.87 | 1 | 0.10 |
| 3 | 77 | 0.3 | 3.35 | 51 | 4.24 |
| 3 | 128 | 0.3 | 3.01 | 6 | 0.45 |
| 4 | 0 | 0.2 | 8.00 | 21 | 2.78 |
| 4 | 21 | 0.2 | 4.62 | 1 | 0.08 |
| 4 | 22 | 0.2 | 2.54 | 15 | 0.63 |
| 4 | 37 | 0.2 | 3.05 | 41 | 2.07 |
| 4 | 78 | 0.2 | 3.52 | 6 | 0.35 |

Table S3: Sevoflurane consumption of the last four cases for anaesthetic gas canister *CH0100009273*.

| Case | Total sevoflurane consumption [g] |
| --- | --- |
| 1 | 13.51 |
| 2 | 17.59 |
| 3 | 17.10 |
| 4 | 5.90 |

# R session information

sessionInfo()

## R version 4.4.0 (2024-04-24)
## Platform: x86_64-unknown-linux-gnu
## Running under: Debian GNU/Linux 12 (bookworm)
##
## Matrix products: default
## BLAS/LAPACK: /gnu/store/in3yw5xrghzmxn29i0mmz5zhpd748mas-openblas-0.3.20/lib/libopenblasp-r0.3.20.so; LAPACK version 3.9.0
##
## locale:
## [1] LC_CTYPE=en_US.UTF-8 LC_NUMERIC=C
## [3] LC_TIME=en_US.UTF-8 LC_COLLATE=en_US.UTF-8
## [5] LC_MONETARY=en_US.UTF-8 LC_MESSAGES=de_DE.UTF-8
## [7] LC_PAPER=de_DE.UTF-8 LC_NAME=C
## [9] LC_ADDRESS=C LC_TELEPHONE=C
## [11] LC_MEASUREMENT=de_DE.UTF-8 LC_IDENTIFICATION=C
##
## time zone: Europe/Berlin
## tzcode source: system (glibc)
##
## attached base packages:
## [1] stats graphics grDevices utils datasets methods base
##
## other attached packages:
## [1] lubridate_1.9.4 gtsummary_1.7.2 english_1.2-6
##
## loaded via a namespace (and not attached):
## [1] highr_0.10 dplyr_1.1.4 compiler_4.4.0
## [4] tidyselect_1.2.1 xml2_1.3.6 stringr_1.5.1
## [7] tidyr_1.3.1 broom.helpers_1.13.0 yaml_2.3.8
## [10] fastmap_1.1.1 R6_2.5.1 generics_0.1.3
## [13] knitr_1.46 backports_1.4.1 forcats_1.0.0
## [16] tibble_3.2.1 bookdown_0.39 rprojroot_2.0.4
## [19] pillar_1.9.0 rlang_1.1.3 utf8_1.2.4
## [22] broom_1.0.5 stringi_1.8.4 xfun_0.43
## [25] bibtex_0.5.1 viridisLite_0.4.2 timechange_0.3.0
## [28] cli_3.6.2 withr_3.0.0 magrittr_2.0.3
## [31] digest_0.6.35 lifecycle_1.0.4 vctrs_0.6.5
## [34] evaluate_0.23 glue_1.7.0 gt_0.10.1
## [37] fansi_1.0.6 rmarkdown_2.26 purrr_1.0.2
## [40] tools_4.4.0 pkgconfig_2.0.3 htmltools_0.5.8.1

# Git commit hash

## [1] "Git commit revision: 98d9fdbd9d84ad66aa9fda950c0bafd74252bab2"
